# The existing therapeutic interventions for orgasmic disorders: recommendations for culturally competent services, narrative review

**Published:** 2015-07

**Authors:** Zahra Salmani, Ali Zargham-Boroujeni, Mehrdad Salehi, Therese K.Killeen, Effat Merghati-Khoei

**Affiliations:** 1 *Student Research Center, School Mursing and Midwifery, Isfahan University of Medical Science, Isfahan Iran.*; 2 *Nursing and Midwifery Care Research Center, Faculty of Nursing and Midwifery, Isfahan University of Medical Sciences, Isfahan Iran.*; 3 *Psychosomatic research center, Isfahan University of Medical Sciences, Isfahan Iran.*; 4 *Addiction Science Division, Medical University of South Carolina, Charleston, South Carolina, USA.*; 5 *Sexual and Family Health Division in Brain and Spinal Injury Research Center (BASIR), Tehran University of Medical Sciences, Tehran, Iran.*

**Keywords:** *Iranian women*, *Orgasm*, *Reproductive health*, *Sexual dysfunction*

## Abstract

**Background:**

In recent years, a growing number of interventions for treatment of female orgasmic problems (FODs) have emerged. Whereas orgasm is a extra biologically and learnable experience, there is a need for practitioners that to be able to select which therapy is the most appropriate to their context.

**Objective:**

In this critical literature review, we aimed to assess areas of controversy in the existing therapeutic interventions in FOD with taking into accounted the Iranian cultural models.

**Materials and Methods:**

For the present study, we conducted an extensive search of electronic databases using a comprehensive search strategy from 1970 till 2014. This strategy was using Google Scholar search, “pearl-growing” techniques and by hand-searching key guidelines, to identify distinct interventions to women's orgasmic problem therapy. We utilized various key combinations of words such as:" orgasm" OR "orgasmic "," female orgasmic dysfunction" OR Female anorgasmia OR Female Orgasmic Disorder ", orgasmic dysfunction AND treatment, “orgasm AND intervention”. Selection criteria in order to be included in this review, studies were required to: 1 employ clinical-based interventions, 2 focus on FOD.

**Results:**

The majority of interventions (90%) related to non-pharmacological and other were about pharmacological interventions. Self-direct masturbation is suggested as the most privilege treatment in FOD. Reviewing all therapies indicates couple therapy, sexual skill training and sex therapy seem to be more appropriate to be applied in Iranian clinical settings.

**Conclusion:**

Since many therapeutic interventions are introduced to inform sexually-related practices, it is important to select an intervention that will be culturally appropriate and sensitive to norms and values. Professionals working in the fields of health and sexuality need to be sensitive and apply culturally appropriate therapies for Iranian population. We further suggest community well defined protocols to screen, assessment and management of women’ sexual problems such as FOD in the Iranian settings.

## Introduction

The notion that culturally competent services should be available to people who are seeking help in sensitive topics such as sexuality has been debated for many decades. Cultural safety and cultural competency are currently important topics for sexual health professionals (1, 2). Culturally competent services will be warranted if therapeutic interventions adapted to the given culture (3).

In management of sexual problems, considerable variation exists in the therapeutic interventions. The range of different therapeutic interventions for women's orgasmic problems has been growing over the recent years, alongside an increasing interest in orgasm related therapies to inform sexual and reproductive health related practices (4-6). While the term "sex therapy" is frequently used to describe how women's orgasmic problems are treated, far more terms are used to describe the therapy of orgasmic problems. The profusion of interventions can mask some of the basic decisive factors in therapy that the different therapeutic interventions share, and also lead to some confusion regarding which therapy is most appropriate in a given culture or context.

No question, there are number of interventions introduced worldwide to treat women's orgasmic problems (7). The psychological and cultural valuation and 'meaning' of orgasm are complex and considerably different in various societies (8). Sometimes the interventions are not efficient because they are not culturally sensitive or appropriate (9, 10). There is a need for researchers and practitioners to be able to identify areas of controversy in the existing therapeutic interventions.

Orgasmic problem is the second most common sexual complaint reported by women. The high prevalence of orgasmic problems and its consequences in one's life lead researchers and professionals to study about etiology and find effective therapies for these problems (11-13). According to the Diagnostic and Statistical Manual of Mental Disorders4^th^ ed. (DSM-IV), Female orgasmic disorder (FOD) is ‘‘Persistent or recurrent delay in, or absence of, orgasm following a normal excitement phase”. The DSM-V adds some explanations to the nature of FOD: “reduced intensity, delay, infrequency or absence of orgasm. The symptoms must last for at least six months and not be related to other physical, mental or relational problem” (14). Epidemiologic research estimated prevalence of orgasmic dysfunction from approximately 20-40% (15, 16). Prevalence of orgasm problem in US and Australia have estimated between 21 to 29 percent (17, 18). Data from a study of 40-80-year-old adults in 29 countries found even higher prevalence of problems with orgasm among women in Asian countries (19). Iranian sexual studies have been restricted to prevalence and related factors of orgasmic disorders. A study with 2526 Iranian women estimated the prevalence of orgasmic disorder about 37% (20). Another descriptive study reported a rate of 21% orgasm disorder among a sample of 1456 Iranian women (21).

FOD a multidimensional problem that influenced by affected personal, sociocultural, religious and political contexts factors (22). Although genetic bases of women’s orgasmic dysfunction are suggested, however, overall pleasure experienced by women through their sexual encounters mostly is contextual and affected by their sexual scripts (23-25). It is necessary to pay attention to those factors at the time of assessment or implementing an intervention (26). This paper does not argue that the various orgasm-related therapies are unnecessary, but rather seeks to draw together and review the full range of therapeutic interventions available to assist future professionals in selecting therapeutic intervention and adopt them into their cultures. Since the existing evidence-based therapies are fused with Western norms, scholars and investigators need to determine whether such treatments are equally effective for other cultures or whether new culturally sensitive therapies are necessary (27).

The primary purpose of this paper is to critically review the existing literature regarding domains of therapies such as pharmacological and non-pharmacological for FODs and to explore areas of cultural controversy in the contexts such as Iran. The secondary purpose of this paper is to suggest implications for clinical practice to be culturally sensitive and competent in Iranian settings. In order to reach out the benefit of this review, we need to answer the following important questions:

1. What make a therapeutic intervention for FOD culturally sensitive in Iranian-Islamic context?
2. Are the existing interventions for FOD adapted and/or tested in the Iranian or similar culture?
3. What possible recommendations for culturally competent services can be made for Iran or other similar contexts?

## Materials and methods

This is a narrative review of literature related to interventions for FODs. This review was approved by the ethic committee of Isfahan University of Medical Sciences in 2014.

**Search Strategy**

Papers which used or discussed relevant interventions were identified by undertaking a high sensitive search using Google scholar, PubMed(including Medline and CINAHL), Embase, Psyc INFO, Cochrane, and hand-searching key journals and guidelines of intervention from 1970-2014. We utilized various key combinations of words such as: "orgasm" OR "orgasmic", "female orgasmic dysfunction" OR “Female anorgasmia” OR “Female Orgasmic Disorder", “orgasmic dysfunction AND treatment”, “orgasm AND intervention” selection criteria in order to be included in this review, studies was required (to: 1 employs clinical-based interventions, 2) focus on FOD. Relevant papers were screened and details of the interventions extracted. All interventions were classified based on their theoretical framework. At first, we found 980 articles based on the key words in the title or abstract. Of 980 articles, 590 were selected for full review. From these articles 98 were selected for the final review if they had introduced at least one therapeutic intervention. After omitting duplicated interventions only 25 distinct interventions were identified. A level of evidence is given to each individual study based on guideline in sexual medicine (2010) (28).

## Results

Overall, the narrative review of available research suggests that interventional therapy of FOD are restricted and often looked controversial. The studies included in this review have introduced non-pharmacological (directed masturbation, sensate focus, cognitive-behavioral therapy, systematic desensitization, sex therapy, couple communication training, educational intervention, sexual health model, hypnotic technique, anxiety reduction techniques, coital alignment technique (CAT), biblotherapy, kegel exercise, orgasm consistency training (OCT), Sex “Aids”, Basic counseling, psychotherapeutic interventions for the individual woman, trauma therapy, modeling, role playing) and pharmacological treatments (testosterone, estrogens, tibolone, sildenafil, bupropion, arginMax). Compared to pharmacological treatment options, non-pharmacological interventions have two main advantages; that is, they do not have negative physical side effects, and they aim at the re-establishment of sexual functioning and the increase of sexual satisfaction beyond the reduction of aim manifestation.

Results showed that psychological interventions are superior to wait-list in improving symptom severity and sexual satisfaction with a significant effect size if conducted in a couple setting sex therapy and sexual skills training were the most frequently studied interventions over the years. Compared to results of this review are not completely consistent, and this discrepancy may be linked to several factors that differed between studies, such as the meanings were used to define and operationalize the term "orgasm".

The results do suggest that direct masturbation can be empirically valid and effective technique for lifelong FOD. Findings from this review put emphasis on meaningful effects of psychological interventions such as marital therapy, sexual skill training and sex therapy on severity and sexual satisfaction in FODs.

The inconsistent use of orgasm-related concepts, for example, subjective and/or reflexive orgasm, pleasurable or not pleasurable sexual encounters are found important limitations of the studies had used the interventions. This does not allow one to reach a certain conclusion. Researcher had used several therapy techniques in FOD by various outcomes implemented (28). Interventional outcomes studies for the treatment of FOD have shown in Table I.

Many of the interventions introduced were implemented in the western cultures which make broad generalizability difficult for practice in FOD. Limited sexual knowledge related to sexuality was found as an important influencing factor in sexual dysfunction among Iranian patients (20, 29, 30). Recent Iranian study examined the effectiveness of one-on-one PLISSIT (P: Permission, LI: limited information, SS: specific suggestion, IT: intensive care) model against the group-based sexual health model in women with sexual dysfunction. In this study PLLISIT model was significantly effective on helping to solve the women's sexual problems. Sexual health model was more efficient than others in motivating women to take action toward solving their own problems by using group members' sexual experiences (31). Another Iranian clinical trial also showed effectiveness of PLISSIT model on sexual dysfunction of women in city of Zanjan (32).

A semi-experimental research was shown on 30 Iranian females aged between 20-40 years old. The females received interventional trainings in 10 sessions within two months. The obtained results revealed that sex therapy with cognitive-behavioral approach would be helpful in treatment of orgasmic disorder (33). Results have been shown the nature of most therapy necessitate that patients completing assigned and exercises, maintaining motivation and resolving sociocultural barriers between clinician visits contributes to positive outcomes of therapy. Techniques that used frequently with other in combination treatment approaches methods were behavioral therapy, asserration training, behavioral analysis, behavioral rehearsal, behavioral sex therapy, history taking, treatment cognitive therapy, anterior fornix erogenous (AFE) zone stimulation, new functional-sexological treatment, sex history, group therapy, sexological examination, sexological interview, mindfulness and yoga practices (4, 34-38).

**Table I:** Interventional outcomes studies for the treatment of female orgasmic dysfunction

**Author and year**	**N**	**Treatment method**	**Results**	**Evidence grade**
Cooper 1970 (39)	50	In vivo desensitization, sex therapy, psychotherapy. No control group	The results indicated a 50% improvement in sexual functioning post-therapy	4
Lopiccolo 1985 (40)	31	Primary and secondary anorgasmia CBT sexual therapy for 5 1-hr session	Increase in orgasm with masturbation /3mo follow-up ;gains maintained /improved	-
Fitchenlibman 1983 (41)	23	Secondary anorgsmia Sexual information relaxation, kegel ex. Direct masturbation, sexual communication training,	No change in orgasm; increase in enjoyment of noncoital sexual caressing	3
Van Lankveld 2001 (42)	9	Orgasmic dysfunction Biblotherapy (including communication skills, sexual education and CBT with telephone support	No improvement in orgasm	-
DeAmicus 1985 (43)	22	Sensate focus, directed masturbation, sensual awareness, communication training, modification of sexual behaviors. No control group	There was a 64% 76% improvement in sexual functioning at post-treatment, and this was maintained at follow-up	2
Heiman 1983 (44)	41	Cognitive behavioral therapy, communication training, directed masturbation, sensate focus, systems conceptualization. Wait-list control group	There was a 15% to 40% improvement in sexual functioning at 3 months follow-up	2
Kilman 1986 (45)	55	Group couples communication skills & sex education vs. group couples sexual skills. These two groups compared to a control group	The results demonstrated 25% improvement in sexual functioning for both treatment groups at post-test. These results were maintained at 6 months follow-up	2
Smith 2008 (46)	25	Evaluated the effectiveness of a group CBT and bibliotherapy program for women with sexual dysfunction. No control group	The women demonstrated significant improvements in their FSFI scores at post-therapy, as well as improvements in most FSFI domain scores	3
Masters 1970 (47)	342	Sensate focus, couples therapy, systematic desensitization, sex education and communication training	There was no control group for this study, but the success rate ranged from 77% - 83%. The follow-up success rate after 5 years was 82%	3
Kuriansky 1982 (48)	19	Systematic desensitization, directed masturbation & assertiveness training for the treatment condition. No control group	The results demonstrated an improvement of 95% in levels of sexual dysfunction a post-therapy, and 84% at two year follow-up	4
Hurlbert 1993 (49)	39	Group intervention including orgasm training was compared to group intervention alone for women with HSD	Both groups of women made improvements in 2 of the 4 sexual behavior measures. The women who received orgasm training showed greater sensual arousal and sexual assertiveness at post-treatment and follow-up	2
McCabe 2001(50)	200	Evaluated the effectiveness of individual CBT for the treatment of sexual dysfunction: 95 males, 105 females. No control group	After therapy, respondents experienced lower levels of sexual dysfunction, more positive attitudes to sex, and fewer aspects of their relationship affected by their sexual dysfunction	3
Sarwer 1997(51)	370	Behavioral sex therapy for 365 married couples with a range of sexual dysfunctions. No control group	Success rate was 65%, with few drop-outs. Amount of sensate focus in last week of therapy was the strongest predictor of success	3
Bilups 2001 (52)	32	Pre- and postmenopausal women with and without female sexual dysfunction 6at-home sessions of clitoral vacuum therapy; 5-15m with or without partner	55%increased orgasm; non-female of sexual dysfunction 42%increase orgasm	-

CBT: Cognitive-behavioral therapy

HSD: Hypoactive sexual disorder

**Table II:** Therapeutic interventions and challenges

**Intervention**	**References**	**Challenges**
Directed masturbation	(40, 53-59)	The act of masturbation is prohibited in Islam, and must be avoided by the believers. It definitely is a sin, and an evil act. Therefore Not possible or feasible to consider an intervention technique for orgasmic dysfunction in Iranian women.
Systematic desensitization	(54, 55, 60) (57, 61-66)	In this way proposed exercises not to be consist of masturbation or other same activity that forbidden in Iranian context of religion.
hypnosis	(55, 65, 67-69)	In Islam this method to issue fatwa from number of mojtahedes legitimate on the condition that not abused and for miss reason .this way sex therapist must be consider all of the aspect of legitimate problem and context of Iranian women in use of this technique.
Couples sex therapy	(47)	This way is acceptance in condition of each couple not reasonable demand and out of the traditional and controversial societies from other.
Biblotherapy	(42, 70-76)	The use of written materials or computer programs, or the listening/viewing of audio/videotapes for the purpose of gaining understanding or solving problems relevant to a person’s developmental or therapeutic needs in Iranian culturally defined.
Modeling	(34)	In Iranian context, sexual relationship is a private issue therefore in this technique not tolerate in Iranian couple context that to be model for others and also view of film in this connection.
Sexual skills training	(71) (53, 77, 78) (79)	Differentiating sexual consent skill or refusal skill that matches with culturally defined
Sexual health model	(80)	The part of masturbation is forbidden in religious context and must be revised and modified in intervention treatment.
Psychodynamic and insight-oriented therapy	(37)	Not cultural restricted in use of this method in privacy condition
Sex Aids	(81-83)	In this method women must be use of vibrator for masturbation or watching the kind of picture or home video that those in Islam not legitimate and this behavior is haram and prohibition. Sexual stimulants are not universal phenomena and can be influenced by cultural diversity.
Orgasm consistency training	(56)	Use of masturbation as a part of this intervention is forbidden.

## Discussion

Having outlined the range of therapeutic interventions identified to address women's disorgasmia, we contrasted these interventions with Iranian clinical culture. Clearly, many of them come from diverse contexts with different cultures and have different approaches to managing orgasmic disorders. Examining these interventions indicates the need for different and culturally sensitive intervention and appropriate approaches to treatment of Iranian women's disorgasmia too.

Recent studies and reviews have argued on the effectiveness of the culturally adapted sexually-related interventions comparing traditional un-adapted therapies. A culturally sensitive intervention would change a specific feature of standard treatment practice (e.g. delivering therapy in the client's own language) (9, 84). Culturally competent services employ interventions "those in which the general treatment approach is determined by the client’s ethnicity or in which many different features are based on cultural considerations” (84).

We identified 98 studies those had introduced at least one of the therapeutic interventions for FOD. The studies varied in the description and assessment of this problem. Few studies have indicated strong impact of context and culture on sexual attitude and behavior and definition of what is considered sexual norms (25, 85, 86). The majority of the interventions were behavioral and cognitive-behavior basically. This finding is not surprising because orgasm is subject to learning and erotic behaviors must be learnt (71, 87, 88). According to Domenech Rodríguez within a pragmatist paradigm, cultural adaptation models were mainly developed to manipulate behavioral and cognitive based interventions (27).

Many of those therapeutic interventions for FODs were in practical and exercise format. The practices may be advantageous because they are more cost-effective, provide effective care for more women, and promote self-efficacy of clients throughout their sexual lives. However, these interventions may be troublesome for women whose cultural values related to sexuality is traditionally scripted and may be especially unacceptable in conservative communities. The Iranian traditional culture of sexuality is constructed in a typical patriarchal society, and this underpins the issues around women and sexuality. Analysis of this ideology shows the impact of men‘s attitudes on women‘s sexual health (89). In such contexts, women do not talk about sex freely or never touch their own sexual organs; and women mainly perceive and experience sexual behaviors in the context of marriage and with their potential husbands (90).

Alternatively, a few studies used interventions in couple therapy format (4, 6, 63). It may be easier to make cultural adaptations in the process of couple therapy. More likely some of culturally competent therapists do some adaptations already. However, such adaptations may be personalized by given therapist so that cultural norms and codes can be manipulated by the therapist based on her/his beliefs and system of values. Therefore, it may be difficult to assess their cultural sensitivity.

For example masturbation method was a substantial method supported by scholars. This exercise is believed to benefit women with orgasmic problems for a number of reasons (91). Inversely some of research showed masturbation has a broad range of effects on the human psyche and psychological defense mechanisms, for example passive aggression, denial, and autistic fantasy (92, 93). Although, masturbation is forbidden in Islam; it is possible some of even Muslim therapists acculturate their therapeutic approaches and suggest the clients’ masturbation as the first line treatment (51). Those of culturally competent therapists would suggest mutual masturbation without explicitly naming it "masturbation". 

The techniques that found culturally problematic showed with their challenges in Table II. We argue that changes in existing interventions for FODs may happen by seriously consideration of cultural values and incorporating spirituality and religion codes. We must consider community members' involvement in development, taking into account their acculturation level, address race, prejudice, and discrimination, and offer strategies to empower the clients. The inclusion of community members in the process of adapting or developing a therapy is seminal to make sexually-related interventions efficient. However, none of the reviewed studies had declared community members' involvement in the process of intervention development.

**Recommendations for interventions**

Our recommendations for therapeutic interventions from this review can be inspired by the Cultural Accommodation Model (CAM) of counseling (94). The key component of CAM is to identify current culturally specific concepts and models from the community to fill in the cultural gaps and accommodate the models to therapists' working approaches. It sounds important to consider the practitioners' cultures and assumptions toward women and sexuality. A range of assumptions can be organized into a spectrum as follows:

- Social dignity of a female patient is much more important than speaking out and intervening her sexual concerns.
- Religiosity has significant effects on Iranian women’s sexual understandings.
- Sexually-related therapies are very open and embarrass the patient.
- The ways Iranian couples negotiate their sexual encounters and the process of consent are unknown and sexual life keeps its secrecy.
- Difficulty and undesirable in questioning Iranian women about their sexual needs and interests.
- Iranian women scarify their sexual pleasure to satisfy their sexual duty through the marital interactions.

Thus, in this spectrum we have highly conservative or solely medical views. Before the 1960s, many women were abashed and anxious about seeking sexual pleasure because of the current social view that a 'good' woman simply tolerated her husband's sexual advances (11, 95). Sexual dysfunction in women may commonly be experienced in the context of psychosocial issue, politics, economics, social class, cultural background, relationship conflicts, and shame or embarrassment about sexuality due to religious beliefs or familial inhibitions (4, 11, 26, 35, 96).

A study comparing multiple ethnic groups revealed that Asian women report more sexual complaints and higher instances of anorgasmia (4). Religiosity has significant effects on Iranian women’s sexual understandings; and that experts working in the fields of treatment of orgasm problem need to be sensitive to the notion that some Muslim women may not speak out their sexuality as an indicator of submission to religious codes, of modesty and of being an idealized Muslim wife (97).

## Conclusion

In this paper, we described a range of therapeutic interventions implemented to address women's disorgasmia. We have examined whether the existing interventions are culturally sensitive or applicable in the Iranian contexts. We found that, while the interventions are profoundly effective in various contexts, there can be clear differences in effectiveness and appropriateness of them in the given contexts such as Iran, where women's sexuality is socially constructed and culturally regulated.

Since many therapeutic interventions are introduced to inform sexually-related practices, it is important to select an intervention that will be culturally appropriate and sensitive to religion. Professionals working in the fields of health and sexuality need to be sensitive and apply culturally appropriate therapies for Iranian women. We suggest community well defined protocols to screen, assessment and management of women’s sexual problems such as FOD in the Iranian settings.

## Note

***This review extracted from Ph.D. thesis. (Zahra Salmani)***
